# Polycyclic Aromatic Hydrocarbons in Sediment and Health Risk of Fish, Crab and Shrimp Around Atlas Cove, Nigeria

**DOI:** 10.5696/2156-9614-9.24.191204

**Published:** 2019-12-06

**Authors:** Oluwafunmilayo O. Olayinka, Adetomi Adeola Adewusi, Olanrewaju Olusoji Olujimi, Adeyinka Adedeji Aladesida

**Affiliations:** 1 Department of Environmental Management and Toxicology, Federal University of Agriculture, Abeokuta, Nigeria; 2 Department of Applied Zoology, Federal University of Agriculture, Abeokuta, Nigeria

**Keywords:** sediment, pink shrimp (*Penaeus notialis*), crab (*Callinectes amnicola*), *Drepane africana*, *Pomadasys jubelini*, PAH, LMW PAHs, HMW PAH

## Abstract

**Background.:**

Polycyclic aromatic hydrocarbons (PAHs) are toxic contaminants and pose health risks to humans and the ecosystem due to their persistence in the environment.

**Objectives.:**

This study determined the concentrations of PAHs in sediment, two species of fish (Drepane africana and Pomadasys jubelini), crabs (Callinectes amnicola) and shrimps (Penaeus notialis) around the Atlas Cove jetty, Lagos, Nigeria.

**Methods.:**

Polycyclic aromatic hydrocarbons were extracted from fish, shrimp, and crabs that were purchased from local fishermen. Sediments were collected at five locations impacted by ship movement and cargo offloading around the Atlas Cove jetty during the period of June to August 2016, using standard methods. Potential toxicity of PAHs in the sediments on the surrounding aquatic organisms was assessed. The PAHs were analyzed using gas chromatography-mass spectrometry. Human health risk assessment was calculated from biota using dietary daily intake and carcinogenic potencies of individual PAH concentrations.

**Results.:**

A total of 17 PAH congeners were detected in sediment samples and ten were detected in biota samples. Concentrations of total PAHs obtained in sediment and fish samples ranged from 2.15 - 36.46 mg/kg and 11.89 - 71.06 mg/kg, respectively. The total PAHs concentration pattern follow the order of P. notialis > C. amnicola > P. jubelini (whole) > D. africana (whole) > D. africana (fillet) > P. jubelini (fillet) > sediment. Concentrations of total PAHs were higher in whole fish than in fillet samples (muscle) in both fish species. High values of PAHs were recorded in the dietary intake (0.10 - 2.33 mg/kg body weight/day) of the organisms. Toxic equivalent quotient values (0.01 to 0.10 mg/kg) were observed to be higher than the screening values (0.0014 to 0.0599 mg/kg). In the muscle of Drepane africana and Pomadasys jubelini, splitting and atrophy of the muscle bundles were observed.

**Conclusions.:**

The concentrations of PAHs in analyzed sediment and organisms were higher than the maximum permissible limit of the United State Environmental Protection Agency (USEPA). Most of the detected PAHs were of petrogenic origin, which is an indication that anthropogenic activities were influencing PAH concentrations.

**Competing Interests.:**

The authors declare no competing financial interests.

## Introduction

Polycyclic aromatic hydrocarbons (PAHs) are ubiquitous in the environment and cause adverse environmental effects. The exploration and exploitation of crude oil and gas resources are a major source of PAHs. Even though improved technologies have been introduced in the petroleum industry, accidents continue to occur, resulting in hydrocarbon pollution of the environment (both land and water) in most oil producing countries, including Nigeria.^1^ Oil from the petroleum industry enters the aquatic environment through gas flaring, disposal of used lubrication oils, washings from oil tanks, leakage from marine vessels, erosion and run off from crude oil polluted lands, refinery effluents, and ruptures from poorly maintained flow lines/installations. Leakage of oil into the environment could be due to sabotage or maintenance and engineering errors. Upon entry into the aquatic environment, the fraction either mixes with water or sinks into the sediment, causing severe damage to benthic organisms. Hydrocarbon pollution impacts fish, crustaceans and mollusks with objectionable odor or flavor, reducing their market value and acceptability.^1^ Major routes of PAH exposure in fish are ingestion of contaminated food and diffusion of water across their gills and skin.^2^ The lipophilic nature and high chemical stability of PAHs make it easier to penetrate biological membranes and accumulate in the fatty tissues of organisms following their uptake. Polycyclic aromatic hydrocarbons have been reported to be readily absorbed by fish and other aquatic organisms on exposure to contaminated materials, thereby reaching elevated levels over that of the surrounding medium.^2^ The bioaccumulation pattern of PAHs vary in aquatic organisms depending on the trophic levels they occupy, however, the organic PAHs physiological burden is dependent on the biotransformative effect of the organisms.^3^ Macrobenthic invertebrates (shrimps, crayfish, mollusks and crabs) are an important and integral part of the aquatic ecosystem and thus reflect any negative effects caused by pollution in the community structure which can affect trophic relationships.^4^ Fish have been reported to be the most sensitive living organism to trace concentrations of toxicants in aquatic habitats.^5^ Shrimps, crabs and fishes are therefore good indicators of pollution in coastal waters and have been used extensively for environmental monitoring.^6^

Polycyclic aromatic hydrocarbons are classified into two main groups, low and high molecular weights, based on their physical and biological properties and number of fused aromatic rings contained in their structure. Light or low molecular weight (LMW) PAHs consist of 2–3 aromatic rings, and heavy or high molecular weight (HMW) PAHs consist of 4–6 rings.^7^,^8^ The HMW PAHs are more persistent and recalcitrant (less readily bio-degraded by indigenous microorganisms) than LMW PAHs and can persist in an aqueous environment and bioaccumulate in aquatic organisms like fish and shrimps, and are more carcinogenic.^9^ The LMW PAHs, although less carcinogenic, also pose toxic risks to many aquatic organisms.^10^ The stability and distribution of PAHs in the natural environment is influenced by the chemical structure, chemical configuration and physical and chemical properties of the aromatic rings.^8^,^11^ The spectrum of PAHs in the water ecosystem, including water, fish, and sediment can provide some information about their emission source. Higher concentrations of LMW PAHs (e.g., acenaphthene, fluorene) are usually related to naturally occurring PAHs, either of petrogenic or pyrogenic origin, while PAHs emitted from combustion processes (pyrolytic origin) often contain elevated concentrations of HMW (e.g., phenanthrene, fluoranthene, pyrene) and fewer LMW PAHs.^11^ Polycyclic aromatic hydrocarbons have received attention due to their potential negative effects on human and ecosystem health. Adverse effects of PAHs have also been observed in marine organisms, which include impairment in growth and development, growth reduction, endocrine alteration, malformations of embryo and larvae and DNA damage.^12–14^

**Abbreviations**
*ERL*: Effect range low
*ERM*: Effect range median
*HMW*: High molecular weight
*LMW*: Low molecular weight
*SV*: Screening value
*TEF*: Toxicity equivalency factor
*TEQ*: Toxic equivalent quotient
*USEPA*: United States Environmental Protection Agency

Aquatic foods are high in nutritive value and thus highly desirable due to their contribution of high-quality protein and low fat content. This attracts consumers to these foods due to their health benefits in addition to their widespread availability and relatively low price, although exposures to toxic chemicals have been a concerning issue for years. In Nigerian markets, fish are one of the most common aquatic organisms available for consumption and reportedly provide over 60% of protein intake.^15^ Food consumption has been identified as an important pathway of human exposure to many contaminants, including PAHs. Therefore, PAH contamination of widely consumed fish species among the populace may have serious health implications as some of these aquatic organisms are caught in water polluted by hydrocarbons.^16^ The objectives of the present study are to determine the concentration of PAHs in sediment samples and in two commercially important fish species, Drepane africana and Pomadasys jubelini, crabs, and shrimps around the Atlas Cove jetty. Additionally, the present study aims to assess the health risk associated with the consumption of fish species and shellfish in the study area.

## Methods

**Figure 1:**
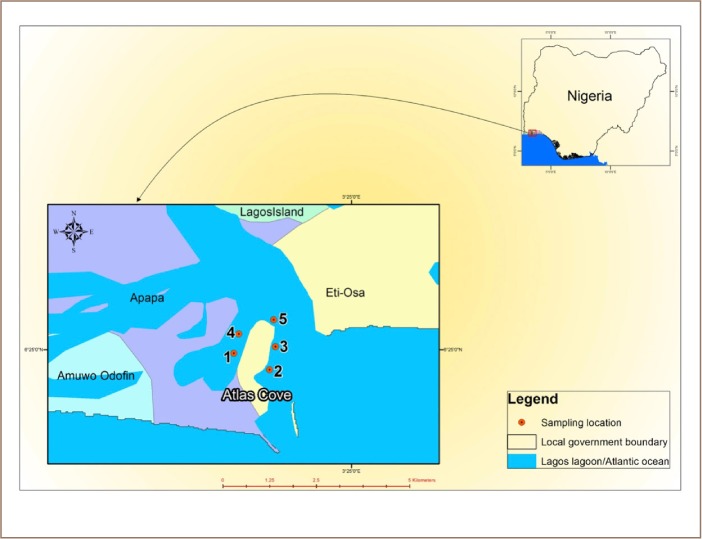
Map of the study area showing sampling points

The study was carried out around the Atlas Cove jetty *(Figure 1)*, which is an offloading and storage depot for imported refined oil prior to distribution to other depots. Oil spillage has been reported in the process of operations in Commodore channel. The channel is approximately 10 m deep at the entrance, 12–15 m deep inward, 0.5 - 1 km wide, and 10 km long. It is tidal, and water from the Atlantic Ocean moves in during high tides and recedes during low tides. Turbulence is very high due to its proximity to the Atlantic Ocean. In addition to oil-related activities that go on around the jetty, it is well known for the movement of shipping vessels along the waterways.

The jetty is located in Lagos State between latitudes 6°21′ N to 6°34′ N and longitudes 3°01′ E to 3°27′ E in the southwestern region of Nigeria. The water around Atlas Cove jetty is brackish (mixture of fresh and marine water). The lagoon plays an important role for the human community due to the large prosperous molluscan and fishing exploitation and commerce. It is also used for artisanal fishing, as a large part of the domestic fish supply in Nigeria comes from inland waters, such as lagoons, and thus provides a means of livelihood for fishermen. However, this industry is being threatened by concentrations of PAHs in this area.

### Sample collection

A total of 45 surface sediment samples were collected in five different locations, at 5 cm depth, around Atlas Cove jetty between June to August 2016. Sediment samples were collected from five different sites, all of which were impacted by anthropogenic activities such as ship traffic and offloading. Sediment samples were collected using sediment grab and transferred onto aluminum foil papers. Twelve (12) samples of each fish species and 20 samples of shrimps and crabs were purchased from local fishermen at the landing site of the study area and transported to the laboratory on ice packs. Upon arrival at the laboratory, the samples were removed from the ice, thawed, and cleaned under running tap water to remove any dirt and then rinsed again with distilled water. The shrimp, crab and fish samples were taxonomically identified using standard reference sources by experts at the Department of Zoology of Obafemi Awolowo University, Ile-Ife Nigeria. Samples were stored separately at −20°C in a freezer.

### Sample identification

**Figure 2:**
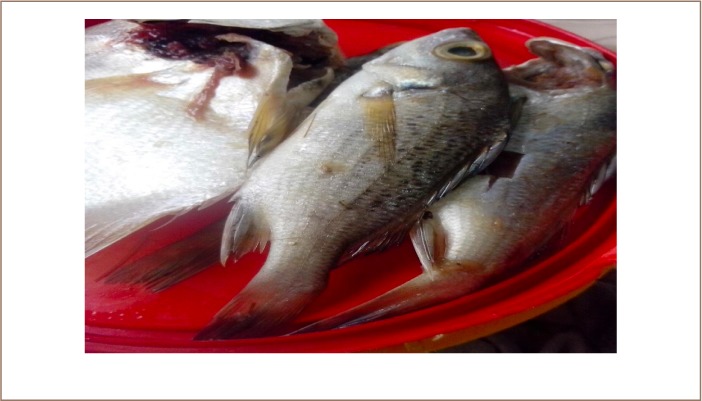
Pomadasys jubelini (grunter) sample from Atlas Cove

**Figure 3:**
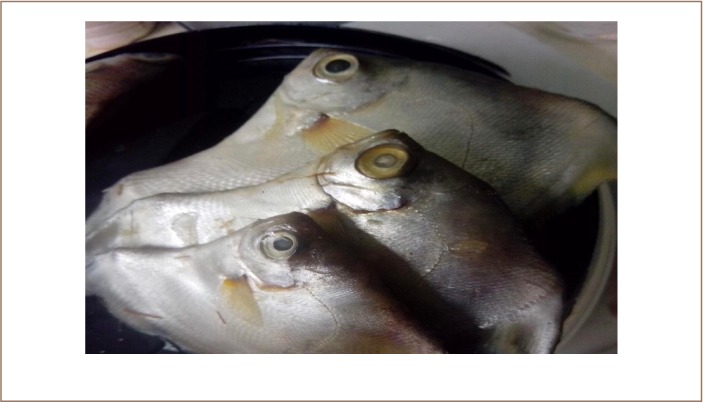
Drepane africana (spadefish) sample from Atlas Cove

**Figure 4:**
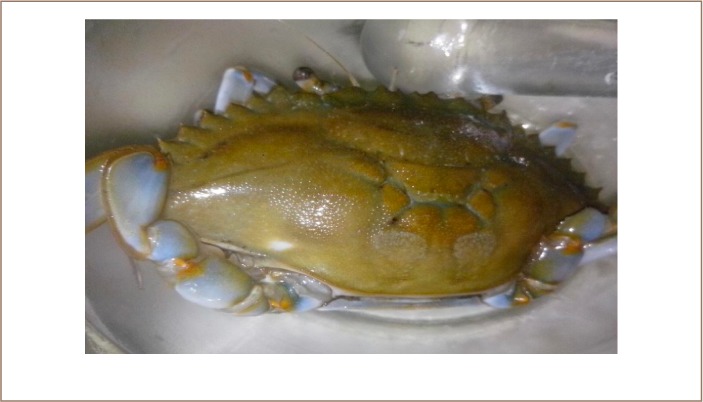
Callinectes amnicola (blue marine crab) sample from Atlas Cove

**Figure 5:**
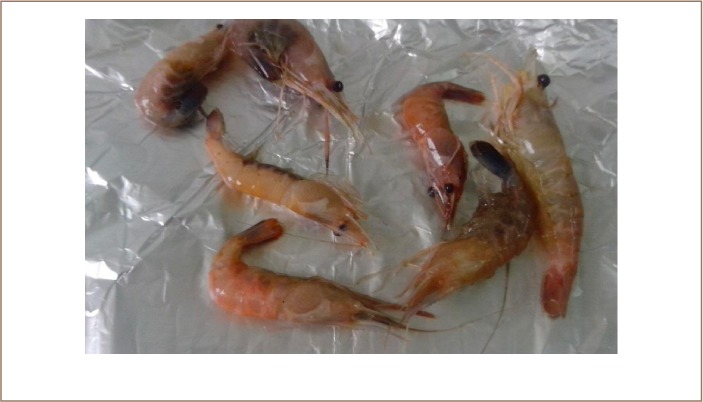
Penaeus notialis (pink shrimp) sample from Atlas Cove

Two species of fishes were identified as Pomadasys jubelini, (common name: grunter) *(Figure 2)* and Drepane africana, (local name: akaraba) *(Figure 3)*. The crab was identified to be the male species of Callinectes amnicola, (common name: marine blue crab) *(Figure 4)*, the shrimp was identified as Penaeus notialis, (common name: pink shrimp) *(Figure 5)*. The sampled fish, shrimp, and crab were identified as either demersal, benthopelagic or pelagic species.

#### Ecology and habitat of Pomadasys jubelini and Drepane Africana

Pomadasys jubelini is found in sandy and muddy bottoms in coastal waters and estuaries and occurs in freshwater and brackish waters. It feeds on fish, small crustaceans, and mollusks.^17^ It is a bottom-living, but periodically pelagic species usually inhabiting littoral waters to about 25 m depth, but has been reported to extend down to about 90 m. It is locally abundant in shallow waters throughout its range. Drepane africana is a neritic, coastal species and occurs in lagoon and estuarine habitats over sandy and muddy substrata between 20 and 50 cm depth. It feeds on fish eggs, benthic invertebrates, and detritus.^18^

#### Ecology and habitat of Callinectes amnicola and Penaeus notialis

Callinectes amnicola (blue marine crab) is a genus of crab from the family Portunidae usually found in the lagoon. The crabs may be considered as euryphagous, feeding on fishes, mollusks, crustaceans, higher plant materials, algae and diatoms. It is found in sand and ocean bottoms.^19^ Penaeus notialis (pink shrimp) is a species of shrimp known to feed on diatoms, green algae, plants materials, as well as crustaceans and fish fragments. It spends a part of its life cycle in open water (ocean), but is mostly found in estuaries, lagoons, open sea and creeks. Its primary habitat (especially adults) is sand, sand- shell or coral- mud bottoms from intertidals.^20^

### Sample pre-treatment

Sediment samples were air dried for several days, after which stones and debris were removed from the samples and then pulverized and passed through a 2-mm mesh sieve to remove other unwanted materials. Samples for PAH analyses were further sieved through 0.5-mm mesh sieve and stored in foil papers until extraction was performed. The fish samples were allowed to thaw, the scales were removed and washed with running water and then distilled water before dissecting and removing the flesh and other parts which were put in sample bottles. Samples included fillets containing only fleshy parts and whole fish, including bone, fleshy parts and organs. The fish samples were weighed using an analytical weighing balance (wet weight) and homogenized with anhydrous sodium sulphate in a mortar with pestle. The mixture was labeled and wrapped in aluminum foil. It was left till the next day to cake, prior to extraction.

### Reference standards

Standard mix solutions of the United State Environmental Protection Agency's (USEPA) 16 priority PAHs, each at 100 μg/L in dichloromethane, were purchased from Sigma-Aldrich (St. Louis, MO, USA). The surrogate standard was a mixture containing naphthalene-d_8_ (N-d_8_), acenaphthene-d_10_ (Ace-d_10_), phenanthrene-d_10_ (Phen-d_10_), chrysene-d_12_ (Ch-d_12_) and perylene-d_12_ (Per-d_12_), which was added to the samples before extraction and used as internal standards for quantification. Stock solutions were used to prepare working standard solutions for calibration and spiking experiments.

### Extraction procedure for PAHs in sediment and aquatic organisms

Polycyclic aromatic hydrocarbons in sediment samples were extracted using a Soxhlet extraction according to the method described by the Association of Official Analytical Chemists.^21^ Five (5) g of each sediment sample was weighed and 5 g of anhydrous sodium sulphate was added to each sample. The samples were placed in a cellulose thimble and extracted for 16 to 24 hours using 150 ml of dichloromethane in a Soxhlet extractor. The extract was concentrated by evaporation overnight in a fume cupboard and covered with a perforated aluminum foil. Polycyclic aromatic hydrocarbons in the biota samples were determined according to the method of USEPA 3540C.^22^

A total of 5 g of each species of shrimp, crab and fish samples that had been previously homogenized with anhydrous sodium sulfate were poured into 100 ml beakers and 40 ml of n-hexane and dichloromethane (1:1 vol/vol) was used as an extracting solvent. The beaker with the content was placed on a magnetic stirrer and shaken for about 25 minutes. The extract was decanted into a clean conical flask, then 20 ml of fresh solvent was added, and the process repeated. The extracts were combined and filtered through a small glass funnel containing a layer of anhydrous sodium sulphate over a plug of glass wool into a receiving conical flask. The extracts were concentrated by allowing to stand overnight in a fume cupboard and covered with perforated aluminum foil.

Sample clean-up was carried out for both sediment and biota using USEPA Method 3630C.^23^ A 600 × 19 mm clean up column was prepared. The hole was blocked with glass wool, 3 g of activated silica gel (60 mesh) was added and the column was topped with sodium sulfate. The column was rinsed by eluting with 20 ml n-hexane and discarded. The concentrated extract was loaded onto the prepared column and eluted with 50 ml n-hexane. The eluates were then concentrated to 1 ml using a rotary evaporator under a gentle stream of pure nitrogen. One (1) ml of the extract was then transferred into a well labeled vial and stored at 4°C prior to gas chromatograph mass spectrometry analysis.

### Instrumental and analytical conditions

Analyses of PAHs were performed for both sediment and biota samples using a gas chromatograph mass spectrometer with selected ion monitoring (Shimadzu QP 2010 gas chromatograph mass spectrometry equipped with AOC 5000 auto injector). The column used was a Varian Factor Four fused silica capillary column (30 m × 0.25 mm × 0.25 μm film thickness) for separating target analytes. Helium was used as the carrier gas at a flow rate of 1.2 mL/min. The sample injector temperature was set at 250°C and 300°C and samples were injected at a volume of 1 mL in splitless mode. An initial column temperature of 60°C was held for 1 min and ramped from 60°C to 200°C at 10°C/min held for 2 min and finally to 300°C at 10°C/min and held for 6 min. The mass spectrometry conditions were set as follows: ionization source: electron ionization at – 70 eV: ion source temperature: 200°C: store mass range m/z 47 - 400 μm. Identification of the individual PAHs was based on comparison of retention time between samples and standard solutions.

### Quality control

The blanks were treated the same way as the samples. Sediment and biota samples were spiked. These fortified matrices were used as calibration standards, and the range of concentrations added to both sediment and biota matrices were used to produce the calibration curves of 20 - 100 mgkg^−1^. The surrogate internal standards were added to the spiked sediment and biota samples at 100 mgkg^−1^. The response factors were then calculated using the response obtained from desorption of a standard solution containing 40 mgkg^−1^ of the 16 PAHs of interest and 100 mgkg^−1^ of each internal standard. Spiked samples were extracted and analyzed. Recovery yields were 75 - 110% and limit of detection for individual PAHs ranged from 0.02 to 30.00 mgkg^−1^ in sediment and biota with a signal to noise ratio of three (3) and limit of quantization of signal to noise ratio of ten (10).

### Human health risk assessment

Toxicological risks associated with PAH concentrations in the biota samples were assessed through comparison of the observed concentrations with regulatory limits and guidelines.

#### Potential human health risk

To assess human health risks from exposure to PAHs through consumption of possibly contaminated biota samples (dietary intake), the dietary daily intake concentrations of PAH's from consumption of contaminated shrimps, crabs and fish species were determined. The dietary daily intake of PAHs in contaminated biota samples were assessed for the adult population using Equations 1, 2 and 3. The daily intake of PAHs from the biota samples was calculated by multiplying the respective PAH concentration in each sample by the consumption rate of an average weight adult (70 kg).

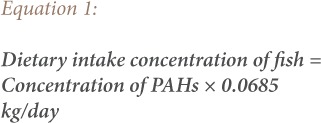


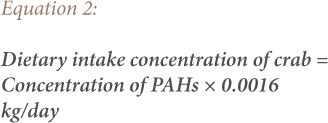


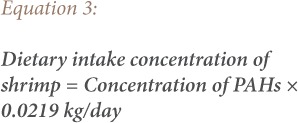
where, 0.0685 kg/day is based on the assumption that an average Nigerian consumes 25 kg of fish per annum and an average of 0.0016 and 0.0219 kg/day of crabs and shrimp, respectively.^5^,^15^

#### Carcinogenic risk assessment of polycyclic aromatic hydrocarbons in biota samples

Cancer risk due to dietary exposure to PAHs in fish was assessed. The carcinogenic potencies of individual PAHs were evaluated by multiplying the PAH concentrations in the sample by the individual toxicity equivalency factor (TEF) as shown in Equation 4.^24^ The toxicity equivalency factor is an estimate of the relative toxicity of individual PAH fractions compared to benzo(a)pyrene. Toxic equivalency factors have been applied as useful tools for the regulation of compounds with common mechanisms of action (e.g. PAHs). The TEFs developed by Nisbet and LaGoy were applied and these values were used to calculate PAH as equivalents for benzo[a]pyrene for a standard adult with 70 kg body weight.^25^ The toxic equivalent quotient (TEQ) was derived by adding the carcinogenic potencies of individual PAHs shown in Equation 5.^24^

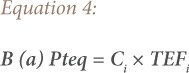


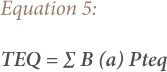
where, B(A)Pteq is the carcinogenic potencies of individual PAHs and C_i_ is the PAH concentration.

The screening value (SV) was calculated as shown in Equation 6, and then compared with the estimated TEQ value to assess the health risk of PAHs associated with consumption of the biota samples. The screening value is referred to as the threshold concentration of chemicals in edible tissue(s) that is of potential risk to consumers. It was calculated using the formula of Nozar *et al*.^5^

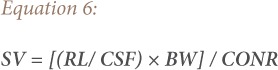
where, RL is the maximum acceptable risk level (10^−5^),CSF is the oral cancer slope factor (7.3 mg/kg/day), BW is body weight (70 kg)*, and CONR is the fish consumption rate.

*Body weight of 70 kg applies to the adult population.^15^

### Histopathological examination

The fish samples were dissected to remove the target organs, while the shrimps and crabs were dissected to obtain the fleshy parts. The gills, fillet, liver of fish and the fleshy parts of the crab and shrimp were put in a separate well labeled bottle, fixed in 5% formalin for at least 48 hours and transferred into a sampling bottle rack. Tissue processing for the histopathological analyses was done according to standard methods.^26^ The tissues were removed from the fixative, and samples were rinsed in tap water for 5 minutes, and dehydrated in ascending ethanol concentrations (70%, 80% and 90% alcohol) for a minimum of 2 minutes each. The dehydrated tissues were cleaned in a wax miscible agent (xylene) for 2 minutes and then embedded in paraffin.

### Tissue sectioning and staining

The fish tissues were then cut into sections approximately 5 μm thick from the block using a rotary microtome (Yamato Kohki, serial no: 75010JO). The cut samples were dried in a hot air oven to remove moisture and each section was mounted on a glass slide. The sections were de-waxed in a wax-miscible agent and rehydrated through descending concentrations of ethanol (90%, 80% and 70% alcohol) for at least 2 minutes each. The sections were then stained with hematoxylin and eosin, after which the tissues were placed in hematoxylin solution for 3 minutes and aqueous eosin for 3 minutes, mounted on a slide and covered with cover slip and labeled.^27^ The tissues were examined, and their microphotographs were taken using a digital binocular compound LED microscope (model MD827S30L series).

### Statistical analysis

The obtained data were subjected to descriptive statistics and analysis of variance. The significant treatment means were separated using Duncan's multiple range tests using the Statistical Package for the Social Sciences software (SPSS) version 19.0.

## Results

Concentrations of PAHs in sediment samples are presented in Table 1. A total of 17 PAHs compounds (naphthalene, 1-methylnaphthalene, 2-methylnaphthalene, acenaphthylene acenaphthene, fluorene, phenanthrene, anthracene, pyrene, fluoranthene, benzo(a)anthracene, chrysene, benzo(b)fluoranthene, benzo(k) fluoranthene, benzo(a)pyrene, indeno(1,2,3-cd) pyrene, benzo(ghi) perylene) were detected in the sediment samples. Concentration of PAHs ranged from 2.15 - 36.46 mgkg^−1^ across the sampling points. Sampling point 5 had the highest (36.46 mg/kg) concentration of PAHs compared to those detected from other points. In the present study, 47.06% of PAHs had 2–3 rings, 23.53% of PAHs had 4-rings, and 29.41% of PAHs had 5–6 rings. The percentage composition pattern of PAHs detected in the sediment samples by number of rings is presented in Table 2. The phenanthrene to anthracene (Ph/An) ratio for sediment samples were 0.65, 2.43 and 1.72 in sampling points 1, 4 and 5, respectively, but were below detection limit in points 2 and 3. The respective values for fluoranthene to pyrene (Fl/Py) ratio were 0.38, 0.66, 0.47 and 1.02 in sampling points 1, 2, 4, and 5, respectively.

**Table 1:** Concentrations of PAHs in Sediment (mg/kg)

PAHs	Sampling locations				

	1	2	3	4	5
Naphthalene	1.03±0.12^a^	1.17±0.01^a^	0.95±0.00^ab^	0.70±0.00^c^	0.66±0.01^c^
1-Methylnaphthalene	0.36±0.03^a^	0.41±0.02^a^	0.31±0.03^a^	0.19±0.02^ab^	0.23±0.00^a^
2-Methylnaphthalene	0.23±0.06^a^	0.27±0.01^a^	0.19±0.03^a^	0.15±0.03^a^	0.12±0.00^a^
Acenaphthylene	1.42±1.11^a^	BDL	BDL	BDL	0.45±0.13^b^
Acenaphthene	0.23±0.19^a^	0.09±0.01^b^	0.03±0.01^b^	0.04±0.01^b^	0.05±0.01^b^
Fluorene	1.05±0.53^a^	BDL	BDL	BDL	BDL
Phenanthrene	2.00±0.19^a^	BDL	BDL	0.17±0.02^c^	0.74±0.07^b^
Anthracene	3.25±0.14^a^	BDL	BDL	0.07±0.01^c^	0.43±0.07^b^
Pyrene	5.43±0.18^a^	0.97±0.04^c^	BDL	0.49±0.00^d^	4.63±2.30^b^
Fluoranthene	2.07±0.08^b^	0.64±0.02^d^	1.63±0.01^c^	0.23±0.02^e^	4.73±2.10^a^
Benzo(a)anthracene	0.76±0.66^b^	BDL	BDL	0.11±0.01^c^	2.48±0.45^a^
Chrysene	BDL	BDL	BDL	BDL	4.97±2.45
Benzo(k)fluoranthene	BDL	BDL	BDL	BDL	3.03±1.44
Benzo(b)fluoranthene	BDL	BDL	BDL	BDL	2.80±1.67
Benzo(a)pyrene	BDL	BDL	BDL	BDL	4.10±1.95
Benzo(ghi)perylene	BDL	BDL	BDL	BDL	3.38±1.89
Dibenzo(a,h)anthracene	BDL	BDL	BDL	BDL	BDL
Indeno(1,2,3-cd)pyrene	BDL	BDL	BDL	BDL	3.67±2.34
∑CPAHs	0.76	-	-	0.11	20.76
Total PAHs	17.82	3.55	3.11	2.15	36.46

^*^Mean concentrations with different superscripts along the same row are significantly different (P≤0.05).

Abbreviations: BDL, below detection limit (detection limit − 0.001 mg/kg); CPAHS, carcinogenic PAH.

**Table 2:** Percentage Composition of LMW and HMW of Total PAHs Detected in Sediment Samples

Number of Rings	% Abundance
2–3 ring PAHs	47.06%
4 ring PAHs	29.41%
5–6 ring PAHs	23.53%

### Polycyclic aromatic hydrocarbon concentrations in Drepane africana and Pomadasys jubelini

Concentration of PAHs in Drepane africana and Pomadasys jubelini samples are presented in Table 3. The concentration of total PAHs in the fillet (i.e. muscle) was 22.67 mg/kg, and 33.97 mg/kg in the whole fish in Drepane africana. For the individual PAH compounds, naphthalene had the highest concentration (28.53±9.19 mg/kg) which was observed in the whole fish sample, while anthracene had the lowest concentration (0.02±0.01 mg/kg) and was observed in the fillet of the fish sample.

**Table 3:** Mean Concentrations of PAHs in Drepane africana and Pomadasys jubelini (mg/kg)

PAHs	Drepane africana		Pomadasys jubeli	

	Fillet	Whole fish	Fillet	Whole fish
Naphthalene	19.17±20.13^b^	28.53±9.19^a^	9.44±3.42^b^	27.60±12.20^a^
1-MethylNaphthalene	1.46±0.86^b^	1.74±0.06^a^	0.76±0.68^b^	2.54±2.04^a^
2-MethylNaphthalene	0.93±0.47^a^	1.05±0.36^a^	0.45±0.37^b^	1.71±1.33^a^
Acenaphthylene	0.07±0.12^a^	0.24±0.04^a^	0.15±0.13^a^	0.33±0.11^a^
Acenaphthene	0.22±0.17^a^	0.14±0.11^a^	0.15±0.13^b^	0.54±0.33^a^
Fluorene	0.34±0.30^a^	0.20±0.17^ab^	0.10±0.11^b^	0.50±0.46^a^
Phenanthrene	0.25±0.21^ab^	0.70±0.62^a^	0.39±0.11^b^	0.91±1.01^a^
Anthracene	0.02±0.01^a^	0.17±0.16^a^	0.08±0.06^a^	0.02±0.01^a^
Pyrene	0.07±0.03^b^	0.52±0.43^a^	0.17±0.05^b^	0.40±0.25^a^
Fluoranthene	0.14±0.11^b^	0.69±0.44^a^	0.20±0.07^b^	0.48±0.35^a^
Benzo(a)anthracene	BDL	BDL	BDL	BDL
Chrysene	BDL	BDL	BDL	BDL
Benzo(k)fluoranthene	BDL	BDL	BDL	BDL
Benzo(b)fluoranthene	BDL	BDL	BDL	BDL
Benzo(a)pyrene	BDL	BDL	BDL	BDL
Benzo(ghi)perylene	BDL	BDL	BDL	BDL
Dibenzo(a,h)anthracene	BDL	BDL	BDL	BDL
Indeno(1,2,3-cd)pyrene	BDL	BDL	BDL	BDL
Total PAHs	22.67	33.97	11.89	35.02

^*^Mean concentrations with different superscripts along the same row are significantly different at P≤0.05.

Abbreviation: BDL, below detection limit (detection limit − 0.001 mg/kg)

Moreover, the concentration of total PAHs in the fillet was 11.89 mg/kg and 35.02 mg/kg in the whole fish in Pomadasys jubelini samples. A total of 10 PAH compounds were detected in the samples. Naphthalene had the highest concentration (27.60±12.20 mg/kg) of individual PAH compounds and was observed in the whole fish sample, while anthracene had lowest concentration (0.02±0.01 mg/kg) and was observed in the whole fish sample.

In the present study, 80% of PAHs had 2–3 rings, 20% had 4-rings, and no PAHs with 5–6 rings were detected in Drepane africana and Pomadasys jubelini samples *(Table 4)*.

**Table 4:** Percentage composition of LMW and HMW of total PAHs detected in Drepane africana and Pomadasys jubelini

Number of rings	% Abundance
2–3 ring PAHs	80%
4 ring PAHs	20%

### Polycyclic aromatic hydrocarbon concentrations in Callinectes amnicola (blue marine crab) and Penaeus notialis (pink shrimp)

Concentrations of PAHs in Callinectes amnicola and Penaeus notialis samples are presented in Table 5. A total of 9 PAHs were detected in C. amnicola, while 10 PAH congeners (naphthalene, 1-methylnaphthalene, 2-methylnaphthalene, acenaphthylene, acenaphthene, fluorene, phenanthrene, anthracene, pyrene, fluoranthene) were detected in P. notialis. All of the LMW PAHs tested for were present except for fluorene, which was not detected in crab. The concentration of total PAHs in the crab sample was 60.30 mg/kg. The highest concentration (46.50±15.66 mg/kg) of individual PAH compounds were obtained in naphthalene and the lowest concentration (0.05±0.02 mg/kg) was obtained in anthracene. The result obtained in this study (6 0030 ng/g) for crab was higher than the result reported for crab; young (1 2138.07 ng/g) and mature crabs with eggs (5629.80 ng/g) collected from Lagos lagoon.^28^ In the present study, 77.78% of PAHs had 2–3 rings, 22.22% of PAHs had 4-rings, and no 5–6 ring PAHs were detected in Callinectes amnicola and Penaeus notialis samples. Concentration of PAHs in shrimp ranged from 0.08±0.13 to 50.65 ± 21.88 mgkg^−1^. The highest concentration was found in naphthalene and lowest concentration was found in acenaphthylene.^29^The percentage composition pattern of PAHs detected in the samples by the number of rings is shown in Table 6.

**Table 5:** Concentrations of PAHs in Callinectes amnicola and Penaeus notialis (mg/kg) Wet Weight

PAHs	Crab	Shrimp
Naphthalene	46.50±15.66^b^	50.65±21.88^a^
1-Methylnaphthalene	7.01±8.38^a^	6.57±7.82^b^
2-Methylnaphthalene	4.70±5.94^a^	4.21±5.27^b^
Acenaphthylene	0.18±0.16^a^	0.08±0.13^a^
Acenaphthene	0.29±0.15^b^	1.31±1.72^a^
Fluorene	BDL	1.78±3.07
Phenanthrene	0.79±1.02^b^	1.65±2.04^a^
Anthracene	0.05±0.02^b^	3.34±2.10^a^
Pyrene	0.27±0.16^b^	0.57±0.59^a^
Fluoranthene	0.51±0.29^b^	0.91±1.20^a^
Benzo(a)anthracene	BDL	BDL
Chrysene	BDL	BDL
Benzo(k)fluoranthene	BDL	BDL
Benzo(b)fluoranthene	BDL	BDL
Benzo(a)pyrene	BDL	BDL
Benzo(ghi)perylene	BDL	BDL
Dibenz(a,h)anthracene	BDL	BDL
Indeno(-1,2,3-cd)pyrene	BDL	BDL
Total PAHs	60.30	71.06

^*^Mean concentrations with different superscripts along the same row are significantly different at P≤0.05.

Abbreviation: BDL, below detection limit (detection limit − 0.001 mg/kg)

**Table 6:** Percentage composition of PAHs in samples of Callinectes amnicola and Penaeus notialis

Number of Rings	% Abundance
2–3 ring PAHs	77.78%
4 ring PAHs	22.22%

### Health risk assessment

The estimated dietary intake values of total PAHs for fillet and whole fish were 1.55 and 2.33 mg/kg body weight/day, respectively. The fish consumption rate was set at 0.0685 kg/day from the annual per capita fish consumption of 25 kg for Nigeria.^15^ The values obtained in this study exceeded the values reported for fish samples from Degele community, Sapele, Nigeria which was reported to be 0.02 - 0.94 mg/kg body weight/day (O. niloticus), 0.02–0.12 mg/kg body weight/day (C. gariepinus), 0.12–0.16 mg/kg body weight/day (H. longifilis) and 0.14–0.58 mg/kg body weight/day (L. falcipinnis), respectively.^30^ The estimated dietary intakes of sampled fish species are shown in Table 7.

**Table 7:** Estimated Dietary Intake of PAHs from Sampled Organisms

Organisms	Estimated dietary Intake (mgkg; body weight/day)
Drepane Afrieana (fillet)	1.55
Drepane Afrieana (whole)	2.33
Pomadasys jubelini (fillet)	0.81
Pomadasys jubelini (whole)	2.40
Crab	0.10
Shrimp	1.56

### Dietary intake of polycyclic aromatic hydrocarbons from Callinectes amnicola and Penaeus notialis

The estimated dietary intake values of total PAHs for crab and pink shrimp were 0.10 and 1.56 mg/kg body, respectively.

The carcinogenic potencies of individual PAHs and TEQ values are presented in Table 8. The TEQ values obtained from TEF values were used to assess the carcinogenicity of PAH contamination in the sampled biota.^31^ The TEQ values of PAHs in the biota samples were 6.08×10^−2^, 1.01×10^−1^, 2.29×10^−2^, 3.55 × 10^−2^, 1.26×10^−2^ and 3.52×10^−2^ mg/kg for crab, shrimp, Drepane africana (fillet), Drepane africana (whole), Pomadasys jubelini (fillet), Pomadasys jubelini (whole), respectively.

**Table 8:** Carcinogenic Potencies of Individual PAHs and Toxic Equivalent Quotient Values

B(a)Pteq values (mg/kg)							
PAHs	TEF	Crab	Shrimp	Drepane africana		Pomadasys jubelini	

				Fillet	Whole	Fillet	Whole
Naphthalene 1-	0.001	4.67×10^−2^	5.06×10^−2^	1.92×10^−2^	2.85×10^−2^	9.44×10^−3^	2.76×10^−2^
Methylnaphthalene 2-	0.001	7.00×10^−3^	6.57×10^−3^	1.46×10^−3^	1.74×10^−3^	7.60×10^−4^	2.54×10^−3^
Methylnaphthalene	0.001	5.00×10^−3^	4.21×10^−3^	9.30×10^−4^	1.05×10^−3^	4.50×10^−4^	1.71×10^−3^
Acenaphthylene	0.001	1.8×10^−4^	8.00×10^−5^	7.00×10^−5;^	2.40×10^−4^	1.50×10^−4^	3.30×10^−4^
Acenaphthene	0.001	2.9×10^−4^	1.31×10^−3^	2.20×10^−4^	1.40×10^−3^	1.50×10^−4^	5.40×10^−4^
Fluorene	0.001	-	1.78×10^−3^	3.40×10^−4^	2.00×10^−4^	1.00×10^−4^	5.00×10^−4^
Phenanthrene	0.001	7.9×10^−4^	1.65×l0^−3^	2.50×10^−4^	7.00×10^−4^	3.90×10^−4^	9.10×10^−4^
Anthracene	0.01	5.00×10^−4^	3.34×10^−3^	2.00×10^−4^	1.70×10^−3^	8.00×10^−4^	2.00×10^−4^
Pyrene	0.001	2.70×10^−4^	5.70×10^−4^	7.00×10^−5^	5.20×10^−4^	1.70×10^−4^	4.00×10^−4^
Fluoranthene	0.001	5.1 ×10^−4^	9.10×10^−4^	1.40×10^−4^	6.90×10^−4^	2.00×10^−4^	4.80×10^−4^
TEQ (values mg/kg)		6.08×10^−2^	1.01×10^−1^	2.29×10^−2^	3.55×10^−2^	1.26×10^−2^	3.52×10^−2^

Abbreviation: B(a)Pteq, carcinogenic potencies of individual PAHs

### Carcinogenic potency of individual polycyclic aromatic hydrocarbons and toxic equivalent quotient

The carcinogenic potencies of individual PAHs and TEQ values are presented in Table 8. The TEFs method was developed to evaluate structurally related compounds and has been applied as a useful tool for the regulation of compounds with a common mechanism of actions (e.g. PAHs). The TEF is an estimate of the relative toxicity of an individual PAH fraction compared to benzo(a)pyrene. The TEF values are used to calculate other PAHs to benzo(a)pyrene equivalents (the most toxic PAHs) for an average adult with 70 kg body weight.^24^ The carcinogenic potency of individual PAHs is represented by the value resulting from the product of the concentration and TEF value of each congener. The TEQ value is the summation of carcinogenic potencies of individual PAH values obtained from a particular sample. It expresses an aggregate measure of toxicity based on a number of contributing compounds.

### Screening value

**Figure 6:**
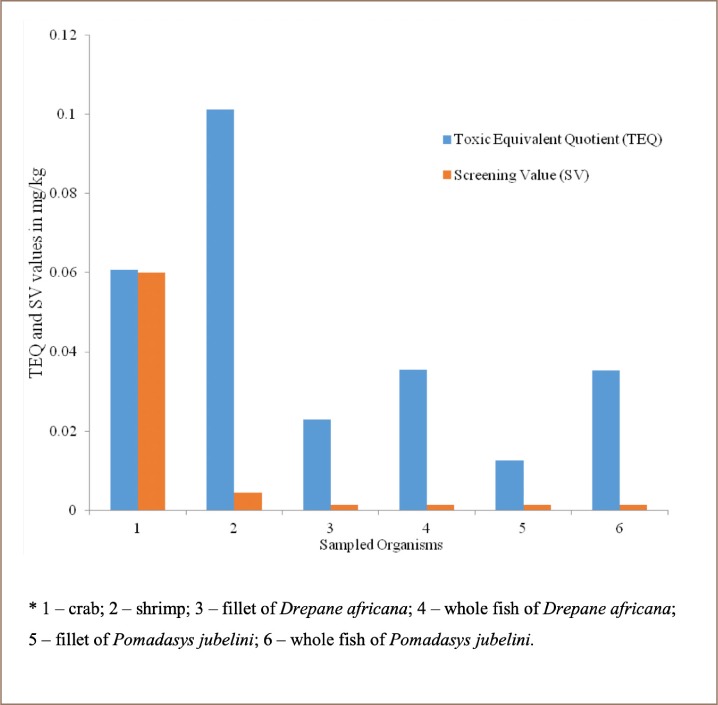
Comparison between TEQ and screening values

The SV was evaluated to assess the health risks posed by PAHs to humans from consuming the sampled organisms. The screening value is defined as the threshold concentration of chemicals in edible tissue that is of potential public health concern.^16^,^32^ An estimated SV of 0.0599, 0.0044 and 0.0014 mg/kg was obtained for crab, shrimp and fish samples, respectively. The resulting TEQ values obtained for the sampled biota exceeded the screening values *(Figure 6)*.

### Histopathological examination

In the present study, the major changes observed in the gills of Drepane africana and Pomadasys jubelini were hypertrophy of the primary lamellae and hyperplasia of the secondary lamellae. Shortening and fusion of the secondary lamellae were observed in Drepane africana, while hyperplasia of the epithelial cells was observed in Pomadasys jubelini. In the muscle of Drepane africana and Pomadasys jubelini, splitting and atrophy of the muscle bundles were observed, while necrosis of the muscle bundles was seen in Pomadasys jubelini only *(Figures 7a–c).* Splitting of the muscle myofibrils was observed in shrimp only, while splitting and atrophy of the muscle bundles were observed in the muscles of shrimp and crab *(Figures 8a–b and 9)*. Changes observed in the liver of Drepane africana and Pomadasys jubelini included necrosis, hepatopancreas and cellular degeneration *(Figure 10a–d)*.

**Figure 7a:**
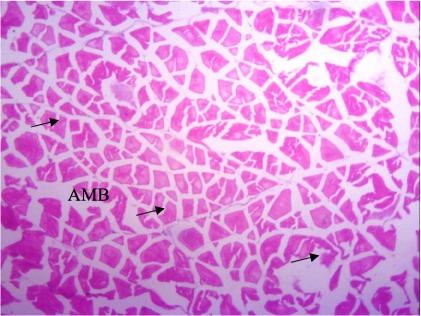
Photomicrograph of muscle section in Drepane africana showing atrophy of muscle bundles (AMB) (Mag. ×100)

**Figure 7b:**
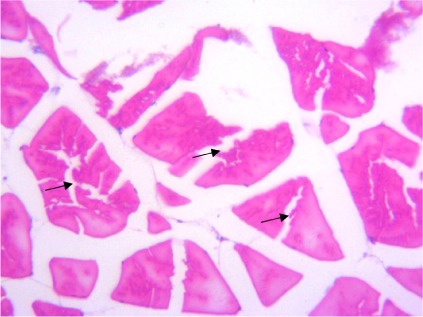
Photomicrograph of muscle section in Drepane africana showing splitting of muscle (SMB) (Mag. ×400)

**Figure 7c:**
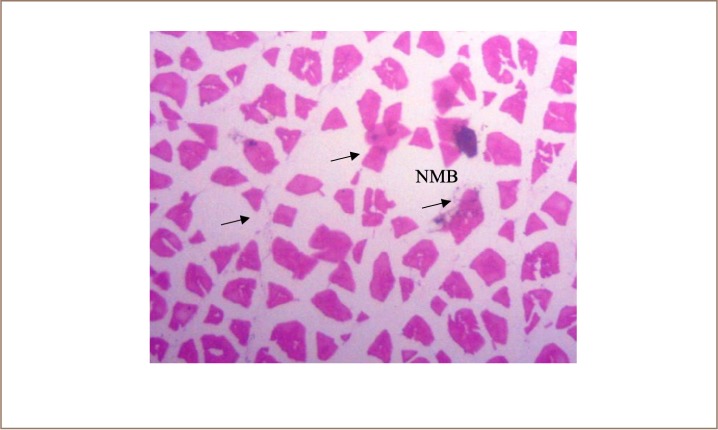
Photomicrograph of muscle section in Pomadasys jubelini showing necrosis of muscle bundles (NMB) (Mag. ×100)

**Figure 8a:**
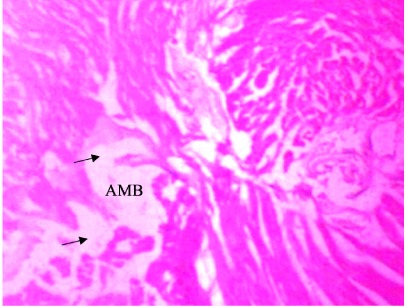
Photomicrograph of muscle section in Penaeus notialis (pink shrimp) showing atrophy of muscle bundles (AMB) (Mag. ×40)

**Figure 8b:**
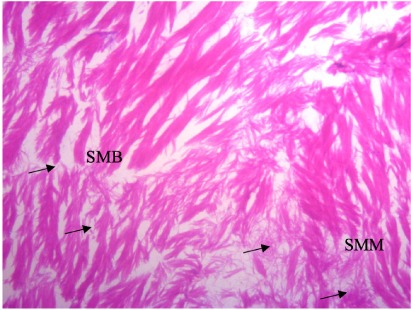
Photomicrograph of muscle section in Penaeus notialis (pink shrimp) showing splitting of muscle (SMB), and splitting of muscle myofibril (SMM) (Mag. ×100)

**Figure 9:**
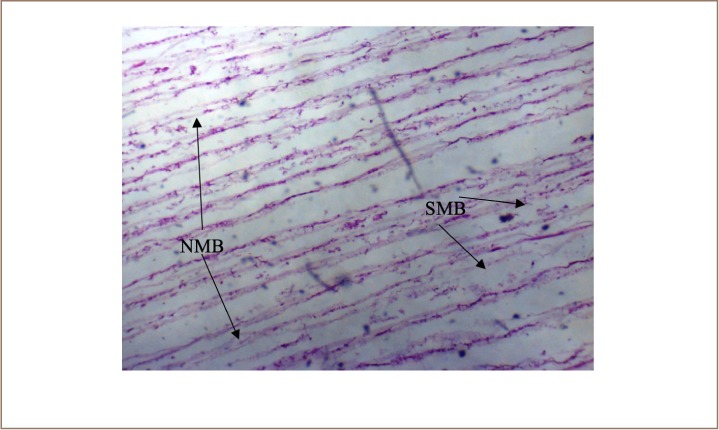
Photomicrograph of muscle section in Callinectes amnicola (blue marine crab) showing necrosis of muscle bundles (NMB) and splitting of muscle (SMB) (Mag. ×40)

**Figure 10a:**
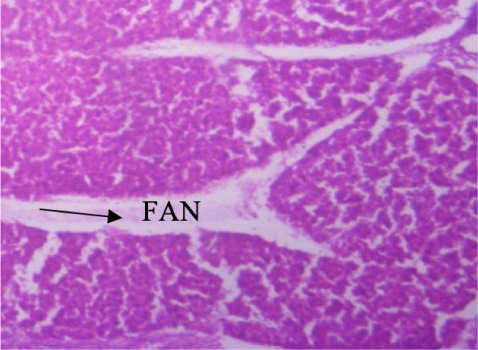
Photomicrograph of liver section in Drepane africana showing focal area hepatopancreas of necrosis (Mag. ×100) degeneration

**Figure 10b:**
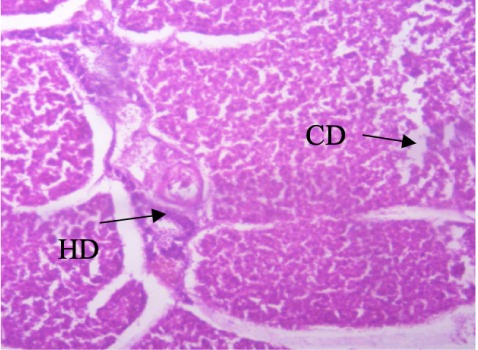
Photomicrograph of liver section in Drepane africana showing degeneration (HD) and cellular (CD) (Mag. ×100) degeneration

**Figure 10c:**
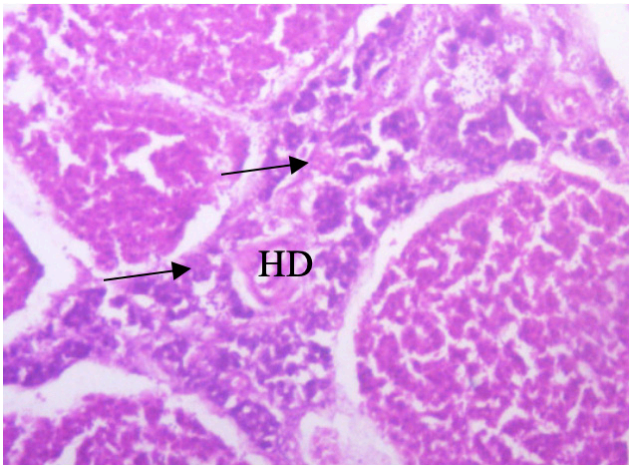
Photomicrograph of liver section in Drepane africana showing focal area hepatopancreas degeneration (HD) (Mag. ×100)

**Figure 10d:**
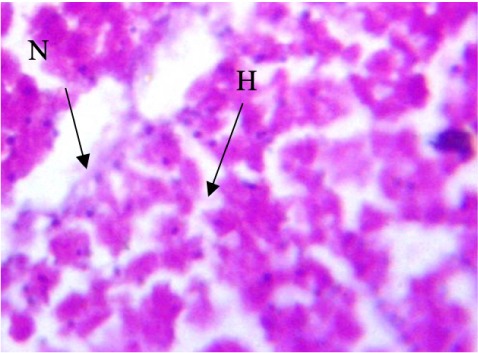
Photomicrograph of liver section in Drepane africana showing the nucleus (N) and hepacytes (H) (Mag. ×100)

## Discussion

The predominance of LMW and HMW PAHs in the sediments reflects the presence of significant combustion products from pyrolytic processes and/or petrogenic sources.^33^,^34^ The preponderance of LMW and HMW PAHs in the sediments indicates the presence of significant combustion products from pyrolytic processes and/or petrogenic sources. 33,34 A sediment quality guideline of 1000 ng/g dry weight total PAHs was designed to protect estuarine fish against several important health effects.^35^ Based on this guideline, the results of this study showed that the concentrations of total PAHs exceeded 1000 ng/g dry weight at all sampling points, indicating that aquatic organisms in the region could be at severe health and environmental risk. Polycyclic aromatic hydrocarbon concentrations in soil/sediment can be classified according to the following categories: >1.0 mg/kg, 0.001 – 1.0 mg/kg and < 0.001 mg/kg for high risk, medium risk, and low risk, respectively.^36^ In the present study, the concentration of PAHs from all sampling points were higher than 1.0 mg/kg, indicating that they presented a high risk. All sediment samples exceeded the USEPA guideline value of 2.5 mg/kg except sampling point 4, which was slightly lower (2.15 mg/kg) *(Table 1)*.

In rural areas, PAHs adsorbed in atmospheric particles can be deposited on the surface of lakes, streams, and oceans by dry or wet deposition, where they could be dispersed by currents and eventually become integrated with sediment. Sediments near urban centers are influenced by atmospheric deposition of PAHs. Other sources of PAHs include storm and sanitary sewer effluents as well as roadway runoff. It was observed that the concentration of PAHs was higher in sediment samples than in water samples. This could be attributed to their hydrophobic tendencies and propensity towards adsorption to particles and solid phases. In addition, they settle and become part of the sedimentary record.

Anthropogenic PAHs originate mainly from combustion of fossil fuels and spillage of petroleum products; from fuel combustion (pyrolytic) or from crude oil (petrogenic). Contamination may be identified by ratios of individual PAH compounds based on peculiarities in PAH composition and distribution patterns as a function of the emission source. Ratios of Ph/An and Fl/Py have been widely used to distinguish petrogenic and pyrogenic sources of PAHs.^37^,^38^ Polycyclic aromatic hydrocarbons of petrogenic origin are generally characterized by Ph/An values > 10, whereas combustion processes often result in low Ph/An ratio < 10. For the Fl/Py ratio, values > 1 have been used to indicate pyrolytic origins and values < 1 are attributed to petrogenic sources. The results from both Ph/An and Fl/Py ratios indicated that PAHs in the Atlas Cove jetty area may originate from both pyrolytic and petrogenic sources. It was observed that the PAHs have pyrolytic sources in sediment samples in all sampling points and this may be attributed to high ship traffic. Potential toxicity of PAHs in sediments on the surrounding aquatic organisms was assessed. The PAHs concentration in the sediments were compared with US National Oceanic sediment quality guidelines *(Table 9)*.^39^ The recommended effect range low (ERL) and effect range median (ERM) target values were used to determine toxic effects in the sampling locations. When PAH concentrations vary between ERL and ERM values, a mild toxic effect is expected. In addition, no negative effect is expected for PAH concentrations lower than ERL values. Table 9 indicates a high probability of risk for organisms that live in sampling locations 1 and 5. Naphthalene, acenaphthene, and fluoranthene exceeded the ERL values, but were within ERM values in both sampling locations, indicating a mild toxic effect. Anthracene exceeded the ERL and ERM values at sampling locations 1 and 5, but was within ERM value at sampling location 5. Anthracene, phenanthrene, pyrene and fluoranthene were below ERL values in sampling location 4. Anthracene, fluorene, phenanthrene, acenaphthylene and pyrene in sampling location 1 and pyrene and benzo(a)pyrene in sampling location 5 exceeded ERM values, suggesting that adverse biological effects such as cancer, reproductive and physiological disorder may occur in fish and mammals.^40^

**Table 9:** Concentrations of PAHs in Sediment Compared with US National Oceanic Sediment Quality Guidelines

PAHs	ERL^39^ (ngg-^1^)	ERM^39^ (nss-^1^)	Sampling locations				

			1	2	3	4	5
Naphthalene	160	2100	1030	1170	950	700	660
Anthracene	85	1100	3250	-	-	70	430
Fluorene	19	540	1050	-	-	-	-
Phenanthrene	240	1500	2000	-	-	170	740
Acenaphthene	16	500	230	90	30	40	50
Acenaphthylene	44	640	1420	-	-	-	450
Pyrene	665	2600	5430	970	1630	490	4630
Fluoranthene	600	5100	2070	640	-	230	4730
Benzo (b) fluoranthene	-	-	-	-	-	-	2800
Benzo(a) pyrene	430	2800	-	-	-	-	4100

Abbreviations: ERL, effective range low; ERM, effective range median.

### Polycyclic aromatic hydrocarbons concentrations in Drepane africana and Pomadasys jubelini

A total of 10 PAH compounds (naphthalene, 1-methylnaphthalene, 2-methylnaphthalene, acenaphthylene, acenaphthene, fluorene, phenanthrene, anthracene, pyrene, fluoranthene) were detected in the samples *(Table 3)*. The less carcinogenic LMW PAHs were detected with naphthalene and its substituent present in the fish samples. The more carcinogenic HMW PAHs (benzo(a)anthracene, chrysene, benzo(k)fluoranthene, benzo(b)fluoranthene, benzo(ghi) perylene, dibenzo(a,h)anthracene, indeno(1,2,3-cd)pyrene) were not detected in the samples of Drepane africana. This result agreed with the report of PAHs in fish samples from the Degele community of Delta state, Nigeria and the bioaccumulation of PAHs in fish and invertebrates of Lagos lagoon.^30^,^41^ A significant (P≤0.05) difference was observed in the total PAH concentrations between fillet and whole fish of the fish species. The percentage composition pattern of PAHs detected in the samples by number of rings is shown in Table 4. The predominance of LMW PAHs as compared to HMW PAHs in the fish samples reflects the presence of significant petrogenic processes.^33^,^34^ The analysis showed that a total of 10 PAHs (8 of which are among the 16 PAHs prioritized by the USEPA) were present in the fish sample. The concentration of PAHs in fish was higher than the water column; this could be due to the fact that PAHs are more readily absorbed by fish than other aquatic organisms on exposure to contaminated materials, thus attaining elevated levels compared to those in the surrounding medium.^6^

The trend for the less carcinogenic LMW PAHs and the more carcinogenic HMW PAHs in Pomadasys jubeli was similar to that observed in Drepane africana presented above. The concentration of total PAHs was lower in the whole fish and fillet of both fish species compared to that of crab and shrimp in the present study. This may be because fish have been reported to have physiological mechanism(s) of rapid PAH biotransformation or depuration and could be influenced by various factors, such as chemical exposure route and time, lipid content of tissues, environmental factors, exposure to multiple contaminants, and differences in species, age, sex, and health conditions of the test animals.^28^ The biotransformation of hydrophobic-containing substances in fish is a major determinant of its toxicity, distribution and excretability.^6^ A large percentage of the world's population depends on seafood, especially fish, to meet their nutritional requirements. In Nigeria, fish provides over 60% of protein intake and is recognized as a very important source of animal protein. Food consumption has been identified as an important pathway of human exposure to many contaminants, including PAHs. Due to the lipophilic nature and high chemical stability of PAHs, they accumulate in the fatty tissues of fish following their uptake.^42^

Exposure pathways of PAHs to fish include bioconcentration from water across their gills, skin and ingestion of PAH-contaminated particulate matter along with food, as PAHs readily adsorb onto particulate organic matter especially soil sediments.^15^ The concentrations of contaminants such as PAHs in fish reflect the state of contamination of the environment.^43^ The observed concentrations of total PAHs in fish in this study indicate high levels of PAHs contamination around the Atlas Cove jetty.

### Polycyclic aromatic hydrocarbon concentrations in Callinectes amnicola (blue marine crab)

Crabs have been reported to have high lipid contents and this increases the chance of absorbing more hydrocarbon molecules, especially those that are not easily degraded or eliminated. The higher concentration of PAHs observed in the present study may be due to the proximity of the sampling points to the Atlas Cove jetty or the movement of ships along the waterways which may serve as a source of pollution. However, PAH concentrations were lower than the values (101.10 - 151.49 μg/g) reported for crabs (Callinectes sapidus) obtained from the coastal area of Ondo State, Nigeria.^29^ The higher levels of PAHs in shrimps are presumably due to food chain bioaccumulation and can be harmful to human health. The concentration of PAHs in crab and shrimp were significant and may have wide environmental implications, affecting bioconcentration in their tissues due to their inability to metabolize PAHs efficiently. The relatively higher concentrations of total PAHs in tissues of shellfish than fish showed the bioaccumulative potency of PAHs by the studied organisms. It may also be due to the fact that shrimps and crabs live directly on and forage in the sediments, whereas fish live up in the water column. Several aquatic organisms such as bivalves, crabs, and shrimps have been reported to bioaccumulate and bioconcentrate organic pollutants in their target organs at levels higher than background concentrations.^44^,^45^ Therefore, they are excellent bioaccumulators of organic and inorganic pollutants. Higher concentrations of total PAHs in banana shrimp and blue crab were compared to Drepane punctata and Pomadasys kaakan, which had lower concentrations of PAHs.^5^

### Health risk assessment

The consumption of Drepane africana at the rate of 68 g/day may induce adverse health effects over time in consumers.

### Dietary intake of *polycyclic aromatic hydrocarbons from* Pomadasys jubelini

The estimated dietary intake values in the present study (0.81 for fillet and 2.40 mg/kg body weight/day for whole fish) were considerably higher in comparison with values reported in other countries of 1.77 - 10.7 ng/kg body weight/day, 626 - 712 ng/day, 13.8 - 16.7 ng/kg body weight/day and 231 ng/day for Mumbai, India, Spain, Korea and Kuwait, respectively.^33^,^46^,^47^

### Comparison of toxic equivalency quotient values

The lowest TEQ value was obtained in the fillet of Pomadasys jubelini. The TEQ values of PAHs in fish species, as reported by Tongo *et al.* were 0.22, 0.005, 0.30 and 0.03 mg/kg in Clarias gariepinus, Tilapia zilli, Ethmalosa fimbriata, and Scomber scombrus, respectively.^24^ The consumption of species with the lowest total mean concentrations of PAHs have a higher potential to cause carcinogenic risk, which agrees with the findings of the present study. Furthermore, the calculated TEQ values were in agreement with the values reported by Iwegbue *et al*.^41^

The results of the present study are in agreement with a study of PAHs contaminants in Chrysichthys nigrodigitatus in Rivers State, Nigeria, where Patrolecco *et al.* reported TEQ values above estimated SVs.^48^,^49^ In addition, higher TEQ values were reported when compared to calculated SVs for PAHs in seafood (fish, crab, and bivalves) in Iran, indicating potential health effects.^5^ However, these results disagreed with reports of lower estimated TEQ values than the SV in studies of PAH concentrations in fish (feral finfish) from a Hong Kong market and the common eel (Anguilla anguilla) from the River Tiber, Italy.^16^,^49^

### Histopathological examination

Histopathological biomarkers are sensitive indicators of subcellular stress in organisms exposed to a range of pollutants over short and long periods of time.^50^ Changes caused by toxic substances can be observed in the gills, such as an increase in pollutant blood diffusion distance as a means for protection.^50^ Fusion of secondary lamellae, which is a result of hyperplasia, brings about a reduction in free gas exchange, thus affecting the general health of the fish. Hypertrophy of the primary lamellae is the enlarging or increase in size of the organ in response to a stressor in the environment. This observation is similar to a report of a high incidence of hyperplasia in Clarias gariepinus and Oreochromis niloticus reported from the Sanyati basin in Lake Kariba, Zimbabwe, as well as hyperplasia in the gills of two species of sturgeons.^50^,^51^ This result is an indication that the fish have been exposed to stressors. Gills are sensitive organs which are easily damaged by numerous pollutants, even at low concentrations. Gills have been reported to perform various vital functions (respiration, osmoregulation, acid-base balance).

Gills have a large surface area in contact with the external environment and are particularly sensitive to chemical and physical changes of the aquatic environment, thereby being a target organ in fish for pollutants carried by water.^51^,^52^ Changes in the structures of these organs as well as in the vital functions performed by the gills were observed due to toxic substances present in the aquatic environment.^53^ The severity of damage depends on the concentration of toxicants and the period of exposure. With prolonged exposure, these lesions could lead to fish mortality. The observed separation of muscle bundles may be due to the initial stimulus of hexachlorocyclohexane, which can induce hyperactivity and excitability in animals, leading to a release of lactic acid and subsequent muscular fatigue. The atrophy observed in the muscle bundles may be due to the exposure to various contaminants.^54^ A study detected changes in muscle tissue of grass carp (Ctenopharyngodon idella) as swelling and necrosis of muscle bundles due to the effect of rice herbicides.^55^ Another study documented physiological disturbances and morphological damage in the muscle tissue of freshwater fish Hoplias malabaricus collected from Ponta Lake in southern Brazil due to bioaccumulation of chlorinated pesticides and PCBs.^56^ Degeneration of muscle bundles with aggregation of inflammatory cells between them and focal areas of necrosis were observed by Gingerich.^57^ The findings of the present study agreed with these previous reports.

The liver is the largest organ in the body and performs several important physiological functions.^58^ Although every tissue has some ability to metabolize chemicals, the liver is the major organ of metabolism or transformation of toxins, making it a target organ that is highly affected by toxins in the system. Necrosis is the death of cells in a tissue or organ caused by disease or injury or as a result of exposure to harmful or toxic contaminants. The necrotic cells are shrunk, and their intercellular attachments are broken.^58^ Vascular dilation may be responsible for the cellular degeneration and necrosis in the liver. When the liver is damaged, excessive blood flows into the liver, blocking the sinusoids. Oxygen deficiency as a result of gill degeneration is the most common cause of cellular degeneration in the liver (*Figures 9a–d*).^59^ The results of this study agreed with previous reports on the effects of different pollutants on fish liver.^54^,^60^

## Conclusions

In the present study, low and high molecular weights PAHs were present in the sediment samples. The concentration of total PAHs in the sediment samples exceeded safe limits, suggesting that the aquatic organisms around Atlas Cove may pose serious human health and environmental risks. The high ratio of LMW PAHs as compared to HMW PAHs suggests that PAH contamination around Atlas Cove may be of natural origin. The concentration of total PAHs in biota samples indicated that PAH contamination from Atlas Cove was relatively high (with dominance of the low molecular weight PAHs). The high concentration of total PAHs present in the organisms indicated that the compounds have bioaccumulated in their tissues and organs over a period of time. The calculated TEQ values were higher than the screening values, indicating potential health effects. Histopathological examination revealed that both fish and shellfish were exposed to high concentrations of PAHs which brought about changes in morphologic structure of these organs, necrosis of the muscle bundles and cellular degeneration.
